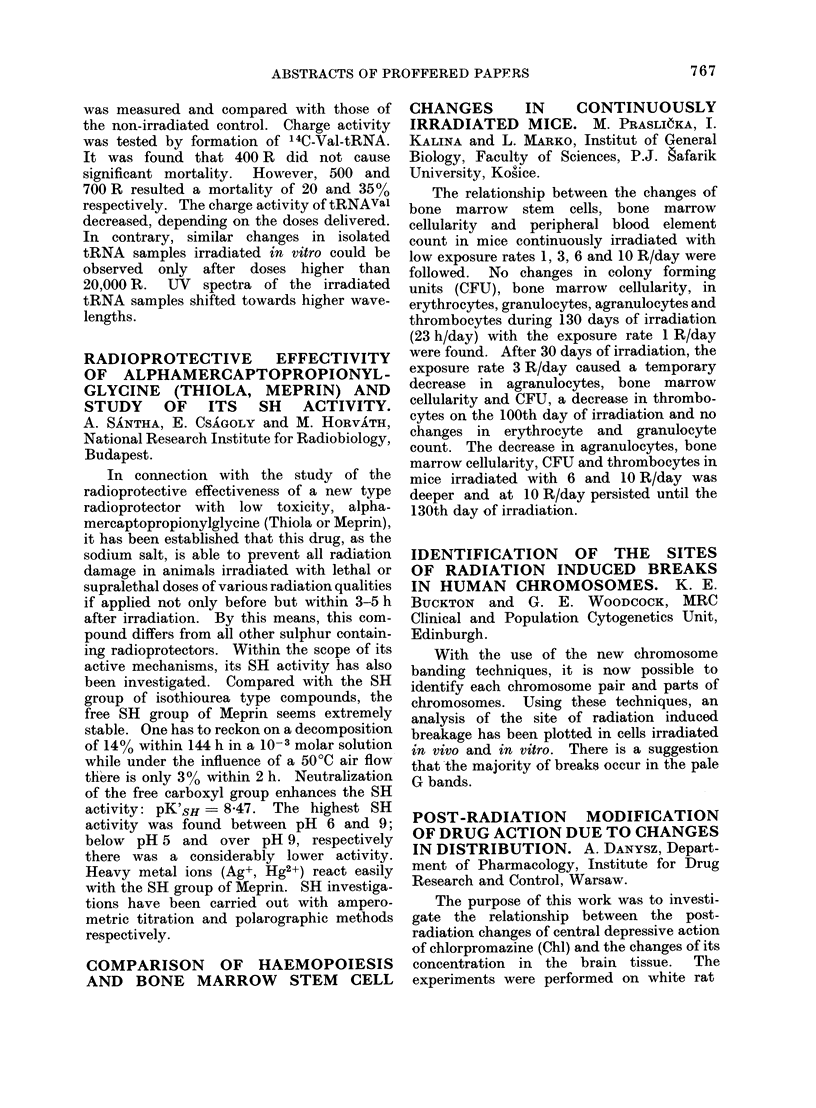# Proceedings: Radioprotective effectivity of alphamercaptopropionylglycine (Thiola, Meprin) and study of its SH activity.

**DOI:** 10.1038/bjc.1975.341

**Published:** 1975-12

**Authors:** A. Sántha, E. Cságoly, M. Horváth


### RADIOPROTECTIVE EFFECTIVITY OF ALPHAMERCAPTOPROPIONYL-GLYCINE (THIOLA, MEPRIN) AND STUDY OF ITS SH ACTIVITY.

A. Sántha, E. Cságoly and M. Horváth, National Research Institute for Radiobiology, Budapest.

In connection with the study of the radioprotective effectiveness of a new type radioprotector with low toxicity, alpha-mercaptopropionylglycine (Thiola or Meprin), it has been established that this drug, as the sodium salt, is able to prevent all radiation damage in animals irradiated with lethal or supralethal doses of various radiation qualities if applied not only before but within 3–5 h after irradiation. By this means, this compound differs from all other sulphur containing radioprotectors. Within the scope of its active mechanisms, its SH activity has also been investigated. Compared with the SH group of isothiourea type compounds, the free SH group of Meprin seems extremely stable. One has to reckon on a decomposition of 14% within 144 h in a $10^{-3}$ molar solution while under the influence of a 50°C air flow there is only 3% within 2 h. Neutralization of the free carboxyl group enhances the SH activity: $pK'_{SH} = 8{\cdot}47$. The highest SH activity was found between pH 6 and 9; below pH 5 and over pH 9, respectively there was a considerably lower activity. Heavy metal ions ($Ag^{+}$, $Hg^{2+}$) react easily with the SH group of Meprin. SH investigations have been carried out with amperometric titration and polarographic methods respectively.